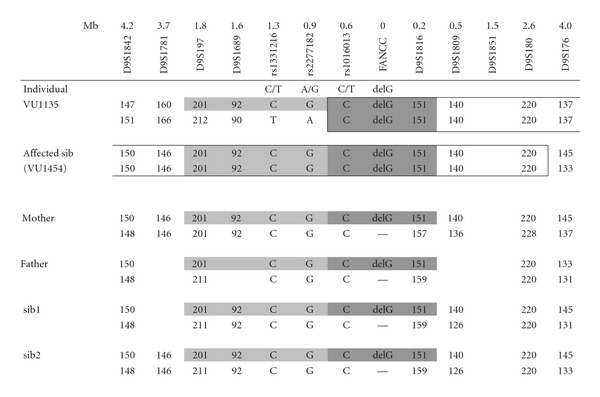# A Dutch Fanconi Anemia *FANCC* Founder Mutation in Canadian Manitoba Mennonites

**DOI:** 10.1155/2012/865170

**Published:** 2012-06-04

**Authors:** Yne de Vries, Nikki Lwiwski, Marieke Levitus, Bertus Kuyt, Sara J. Israels, Fré Arwert, Michel Zwaan, Cheryl R. Greenberg, Blanche P. Alter, Hans Joenje, Hanne Meijers-Heijboer

**Affiliations:** ^1^Department of Clinical Genetics, VU University Medical Center, P.O. Box 7057, 1007 MB Amsterdam, The Netherlands; ^2^Department of Pediatrics and Child Health, University of Manitoba, 675 McDermot Avenue, Winnipeg MB, Canada R3E 0V9; ^3^Department of Cardiac Sciences, St. Boniface General Hospital, 405 Tache Avenue, Winnipeg MB, Canada R2H 2A6; ^4^Medical Diagnostic Center Amstelland, P.O. Box 8018, 1180 LA Amstelveen, The Netherlands; ^5^Department of Pediatrics, VU University Medical Center, P.O. Box 7057, 1007 MB Amsterdam, The Netherlands; ^6^Department of Pediatric Oncology/Hematology, Erasmus MC, Sophia Children's Hospital, P.O. Box 2060, 3000 CB Rotterdam, The Netherlands; ^7^Clinical Genetics Branch, Division of Cancer Epidemiology and Genetics, Department of Health and Human Services, National Cancer Institute, 6120 Executive Boulevard, 1Executive Plaza South, Room 7020, Rockville, MD 20852-7231, USA

## Abstract

Fanconi anemia (FA) is a recessive DNA instability disorder associated with developmental abnormalities, bone marrow failure, and a predisposition to cancer. Based on their sensitivity to DNA cross-linking agents, FA cells have been assigned to 15 complementation groups, and the associated genes have been identified. Founder mutations have been found in different FA genes in several populations. The majority of Dutch FA patients belongs to complementation group FA-C. Here, we report 15 patients of Dutch ancestry and a large Canadian Manitoba Mennonite kindred carrying the *FANCC* c.67delG mutation. Genealogical investigation into the ancestors of the Dutch patients shows that these ancestors lived in four distinct areas in The Netherlands. We also show that the Dutch and Manitoba Mennonite *FANCC* c.67delG patients share the same haplotype surrounding this mutation, indicating a common founder.

## 1. Introduction

Fanconi anemia is an inherited chromosomal instability disorder associated with developmental abnormalities, bone marrow failure, and a predisposition to cancer. A characteristic feature of FA cells is their hypersensitivity to DNA cross-linking agents such as diepoxybutane (DEB) and mitomycin C (MMC). This feature has been used to assign FA cells to different complementation groups. Currently, 15 different complementation groups and their associated genes have been identified. Of these, 14 have an autosomal recessive, and one has an X-linked, mode of inheritance [1–3]. The majority of FA patients belong to complementation group FA-A (65%) followed in frequency by FA-C (10%) and FA-G (10%) [4].

The incidence of FA is approximately 1 in 130,000 live births, with a carrier frequency of approximately 1 in 181 [5]. In some ethnic groups, however, the incidence is much higher due to genetic isolation and a founder effect. For example, founder mutations in the *FANCA* gene have been found in several populations including the South African Afrikaners, Spanish Gypsies, and Moroccan Israeli Jews [6–8]. Furthermore, sub-Saharan Blacks and Japanese carry founder mutations in the *FANCG* gene [9, 10]. In addition, *FANCC* c.456 + 4A > T (also known as IVS4 + 4A > T) is a previously identified founder mutation in the *FANCC* gene in the Ashkenazi Jewish population [11, 12]. Remarkably, the *FANCC* c.456 + 4A > T mutation has a severe phenotype in Ashkenazi Jews, but a milder phenotype in Japanese FA patients, suggesting the presence of unidentified modifying factors [13]. The majority of Dutch FA patients belong to complementation group FA-C, with c.67delG (also known as 322delG) being the predominant mutation ([14], personal communication H. Joenje). In this paper, we report 15 patients of Dutch ancestry and a large Canadian Manitoba Mennonite kindred harbouring the *FANCC *c.67delG mutation. The presence of the *FANCC *c.67delG mutation in this kindred together with the fact that the Mennonites arose in The Netherlands around 1550–1600 AD suggested a common founder for the Dutch and Mennonite c.67delG mutation. We demonstrate that the Dutch and Manitoba Mennonite *FANCC* c.67delG patients do, in fact, share the same haplotype surrounding this mutation, indicating a common genetic origin.

## 2. Materials and Methods

### 2.1. Dutch Patients

All 15 patients with the *FANCC* c.67delG mutation lived in The Netherlands except VU449, VU654, and VU911 who lived in the United Kingdom, Northern France, and Canada, respectively and had Dutch grand and great grandparents. The FA diagnosis was based on clinical symptoms suggestive of FA, in combination with a positive result from a chromosomal breakage test using a standard DNA cross-linking agent. Classification as an FA-C patient was based on complementation studies or sequencing analysis.

Genomic DNA was isolated from fibroblasts, blood, or lymphoblastoid cell lines from previously diagnosed *FANCC* c.67delG patients using the Qiagen Blood mini kit (Qiagen, Venlo, The Netherlands).

### 2.2. Canadian Manitoba Mennonite Family

The proband of this family was diagnosed with FA at the age of five years, when she presented with bone marrow failure. At birth, there was documented intrauterine growth retardation, joint contractures, and a ventricular septal defect. At the time of presentation, the proband was also noted to be of small stature and to have a triangular facies and multiple café-au-lait macules. She died at the age of six years from complications following allogeneic bone marrow transplantation from her HLA-identical sibling. A younger sibling (VU1454) was subsequently diagnosed with FA and was found to be homozygous for the *FANCC* c.67delG mutation. She had normal growth parameters at birth and no noted FA-associated anomalies except for the development of café-au-lait macules.

A field trip was conducted to the rural community in which this family lived, and family members were given an FA information session. Forty-five members of the extended family consented to participate in this study. Clinical histories were obtained, and cheek swabs were collected for genotyping. Minors who provided assent or whose parents consented on their behalf were also included in the study.

### 2.3. Genotype Analysis

Patients and family members were genotyped using fluorescently labelled microsatellite markers and single-nucleotide polymorphisms (SNPs) in an 8 Mb region surrounding the *FANCC* gene. Markers used were the CA repeat markers D9S1842, D9S1781, D9S197, D9S1689, D9S1816, D9S1809, D9S1851, D9S180, and D9S176. Amplification of the CA repeat markers was performed with the GeneAmp PCR system 9700 (Applied Biosystems, Foster City, CA, USA). Samples were analyzed on ABI 3730 or ABI 310 DNA Analyzer (Applied Biosystems, Foster City, CA, USA). SNPs used were rs1331216, rs2277182, and rs1016013. Amplification of the SNPs was performed with the GeneAmp PCR system 9700, and the PCR products were purified using a SAP/EXO treatment (Amersham Biosciences, Uppsala, Sweden) according to manufacturer's instructions. Sequencing was performed with the Big Dye Terminator v3.1 Cycle Sequencing kit (Applied Biosystems, Foster City, CA, USA). Samples were analyzed on an ABI 3730 DNA Analyzer (Applied Biosystems, Foster City, CA, USA).

Genotyping of the Canadian patient and family members for the *FANCC* c.67delG mutation was performed using standard methods (GeneDx, Inc., Gaithersburg, MD, USA). In brief, an allele-specific assay was designed to detect the presence of the single base deletion, and all samples showing the heterozygous presence of the deleted G nucleotide were sequenced bidirectionally to confirm the findings.

## 3. Results

Eleven of the 15 FA-C patients of Dutch ancestry analyzed in this study were homozygous for the c.67delG mutation, and 4 were compound heterozygous, each with a different second mutation. Using polymorphic microsatellite markers and SNPs, the smallest common haplotype in the 26 alleles harbouring the c.67delG mutation was determined to be 0.8 Mb, covering the region from rs1016013 until D9S1816 (Table 1(a)). A larger common haplotype of 2 Mb (D9S197-D9S1816) was found in 23 alleles. The 3 alleles responsible for the decrease of 1.2 Mb in the common haplotype belonged to 3 sibs (VU1134, VU1135, and VU1136) homozygous for the c.67delG mutation. The presence of two partially differing haplotypes in these homozygous c.67delG patients was confirmed in the parents (results not shown). The identical haplotype surrounding the c.67delG mutation indicates that all 15 FA patients have coinherited this stretch of DNA from a common ancestor. The expected size of a co-inherited stretch of DNA can be estimated by the equation 200/(number of meioses), which is based on the assumption that recombinants occurring in meioses from the first common founder on either side of the mutation are uniformly distributed over the interval of 0 to 100 Mb [15]. This indicates that the descent tree of the 15 FA patients, up to the common founder, given a shared common stretch of DNA of approximately 0.8 Mb, counts about 250 meioses. This corresponds to about 10–20 generations and a common founder living in the 16–18th centuries.

**Figure 1:**
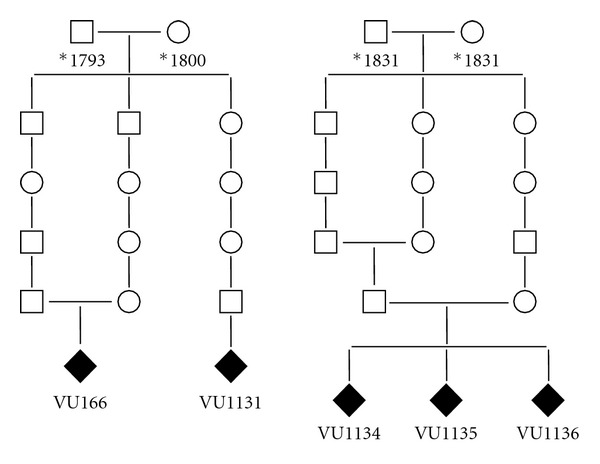
Pedigrees showing consanguinity for patients VU166 and VU1134/35/36. Common ancestors of patients VU166 and VU1131 were found living around 1800. *year of birth.

**Figure 2:**
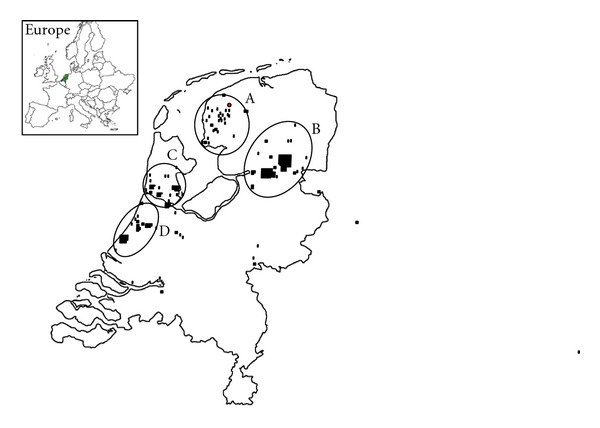
Domicile of the ancestors of patients. VU811 (area A), VU166, VU1131, and VU1134/35/36 (area B), VU239 (area C), and VU806 (area D). Ancestors of patients VU001 and VU002 lived in area B, C, and D. Ancestors of patient VU158 lived in areas C and D.

Genealogical investigations into the ancestors of these patients only retrieved 6 or 7 generations from the archives, and therefore it was not possible to reveal the common ancestor. However, for patient VU166 and patient VU1131, a common ancestor couple was found living around 1800 AD, and consanguinity was shown for patients VU166, VU1134, VU1135, and VU1136 (Figure 1). Our genealogical investigations also showed that most of these 6th and 7th generation ancestors lived in 4 distinct areas in The Netherlands (Figure 2), further supporting the possibility of a single common ancestor. 

Forty-five individuals in the Manitoba family were genotyped for the *FANCC* c.67delG mutation. Eighteen of the 45 (40%) participants were heterozygous for the *FANCC* c.67delG mutation, and no new homozygotes were identified. The medical histories collected from kindred members did not reveal any early-onset cancer, but did document several other disorders already known to be overrepresented in the Manitoba Mennonite population, including severe combined immunodeficiency (SCID) (C.R. Greenberg, unpublished observation) and hypertrophic cardiomyopathy (HCM) [16]. Consanguinity could not be shown in five generations of ancestors. Haplotype analysis was done on the affected sib, two unaffected sibs, and the parents of the proband; it revealed the same common haplotype as found in the Dutch* FANCC* c.67delG patients (Table 1(b)).

## 4. Discussion

This paper shows that c.67delG in *FANCC* is a founder mutation that has probably originated in The Netherlands. This Dutch mutation is also present in a large Mennonite kindred with members living in the province of Manitoba, Canada, and in the United States. The Mennonites are a religious denomination named after one of its founders, Menno Simons, who was born in 1496 in The Netherlands in the province of Friesland, which is the area where the ancestors of the Dutch c.67delG patient VU811 lived. They fled religious persecution over the centuries to the east and Russia, and from there to other parts of the world, and many settled in western Canada during the 19th century [17]. It is therefore likely that the Manitoba Mennonite FA-C patients in this study were descended from the same common ancestor as the Dutch patients in this study. This founder must have lived more than 200 years ago, since genealogical investigation could not identify the common ancestor among the ancestors living after 1800 AD.

Even though the c.67delG mutation is generally thought to be a mild mutation probably due to FAC polypeptide isoforms retaining partial function leading to a mild disease phenotype [18], clinical variability can be seen. The affected Mannitoba Mennonite siblings showed apparent divergent phenotypes, but neither had skeletal abnormalities, a characteristic of the mild disease phenotype associated with the c.67delG mutation.

Based on the high degree of consanguinity, the presence of the Dutch founder mutation, and the geographic origin being The Netherlands, we suspect that the carrier frequency for this c.67delG mutation in the North American Mennonite population is higher than expected for a rare recessive trait. An unselected and unbiased survey would be required in order to determine the actual *FANCC* carrier frequency in this Mennonite population. Several genetic disorders are well known to be overrepresented in this population, but FA has not previously been included in this list. Both HCM and 17 alpha-hydroxylase deficiency have previously been shown to be caused by Dutch founder mutations [19–21] among the Mennonites. Since present generations continue to marry according to traditional custom, we may see an increased frequency of FA-C patients in future generations. Therefore, Mennonite communities should be offered comprehensive genetic counselling and carrier testing for FA.

## Figures and Tables

**Table 1a:** (a)

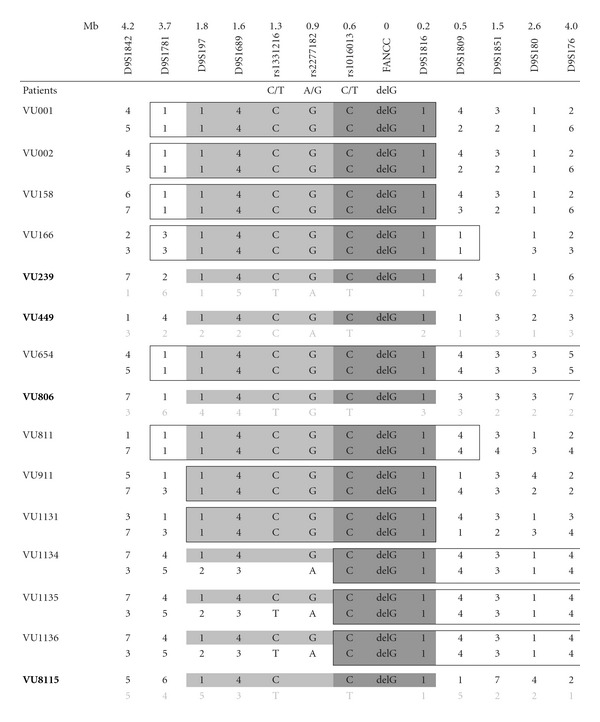

**Table 1b:** (b)